# The Use of Invisalign® System in the Management of the Orthodontic Treatment before and after Class III Surgical Approach

**DOI:** 10.1155/2016/9231219

**Published:** 2016-06-27

**Authors:** Renato Pagani, Fabrizio Signorino, Pier Paolo Poli, Pietro Manzini, Irene Panisi

**Affiliations:** ^1^Maxillofacial Surgery Unit, Carlo Poma Hospital, Strada Lago Paiolo 10, 46100 Mantua, Italy; ^2^Specialization School in Maxillofacial Surgery, University of Milan, Via Commenda 10, 20122 Milan, Italy; ^3^Department of Dental Implants, Maxillofacial Surgery and Odontostomatology Unit, Fondazione IRCCS Cà Granda, University of Milan, Via Commenda 10, 20122 Milan, Italy; ^4^University of Milan, Via Commenda 10, 20122 Milan, Italy

## Abstract

The approach to skeletal dysmorphisms in the maxillofacial area usually requires an orthodontic treatment by means of fixed appliances, both before and after the surgical phase. Since its introduction, Invisalign system has become a popular treatment choice for the clinicians because of the aesthetics and comfort of the removable clear aligners compared with the traditional appliances. Therefore, the aim of the present report was to illustrate the management of a malocclusion by means of Invisalign system associated with the traditional surgical technique. The present paper shows a case of a 23-year-old male patient characterized by a Class III malocclusion with lateral deviation of the mandible to the left side and cross-bite on teeth 2.2, 2.3, and 2.4. Invisalign system was used during the pre- and postsurgical phases rather than fixed appliances. The posttreatment cephalometric analysis emphasized the stability of the dental and skeletal symmetry corrections, occlusion and functional balance, over a 6-year follow-up. The results achieved at the end of the treatment showed how Invisalign can be effective in the management of the orthodontic phases in orthognathic surgery. The follow-up after 6 years emphasizes the stability of the treatment over time.

## 1. Introduction

For many patients, the surgical treatment of Class III malocclusion represents the only available therapy. It requires time, due to not only the diagnostic and planning phases, but also the duration of the treatment itself. For this reason, in these patients, the motivation is an important issue that must not be underestimated [1]. One of the most relevant problems, particularly with regard to adult patients, is represented by the need to undergo orthodontic therapy for several months. The aesthetic problem, associated to the worsening of oral hygiene conditions, may discourage many patients even before the beginning [2]. Invisalign system (Align Technology, Inc., San Josè, California, USA) could represent a suitable solution to solve such problematic [3–5]. It consists in a series of transparent aligners that are able to perform orthodontic movements without compromising the aesthetic of the smile. Hence, the purpose of this paper was to show the effectiveness and the advantages of this device in a surgical treatment of a Class III malocclusion. 

## 2. Case Presentation

### 2.1. Diagnosis

**Figure 1:**
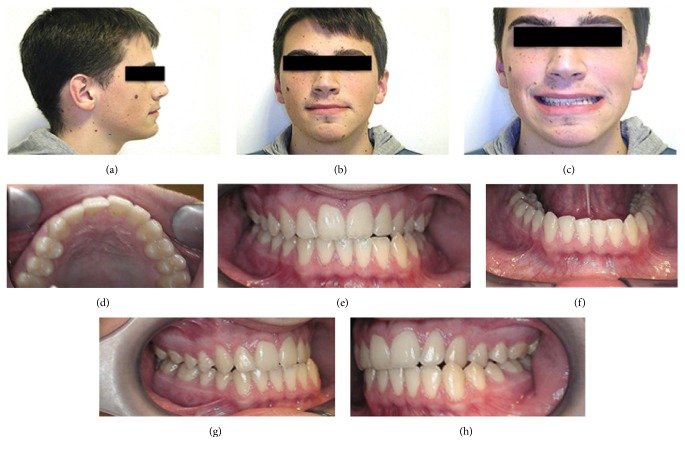
Preoperative clinical evaluation. ((a), (b), (c)) Extraoral evaluation; ((d), (e), (f), (g), (h)) intraoral evaluation.

**Figure 2:**
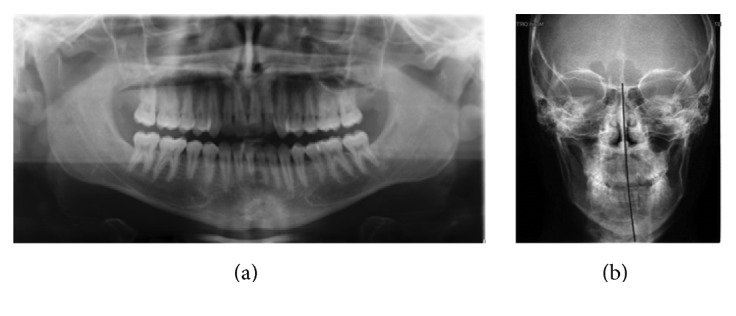
Preoperative radiological evaluation. (a) Orthopantomograph; (b) teleradiography of the skull in a posteroanterior projection.

**Figure 3:**
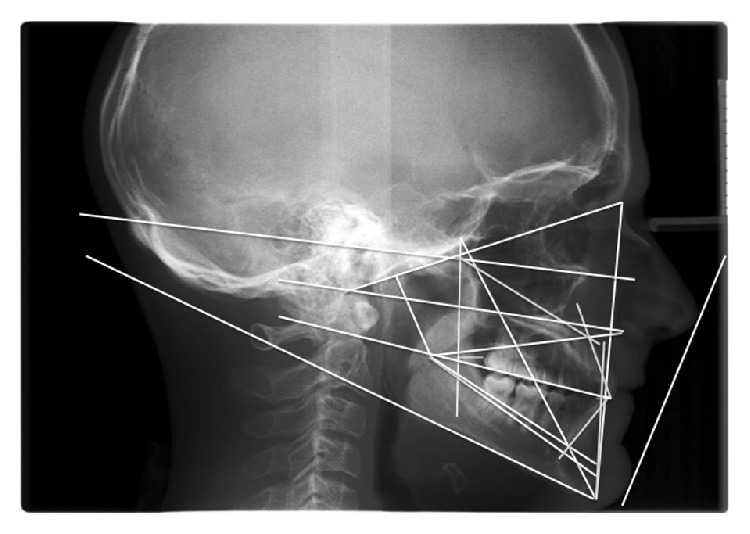
Preoperativelaterolateral teleradiography of the skull and cephalometric analysis.

**Figure 4:**
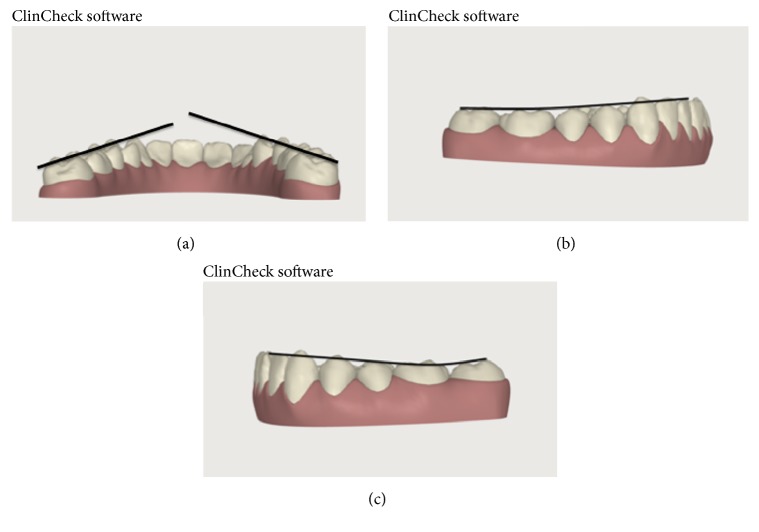
(a) Curve of Wilson; (b) curve of Spee in the right side; and (c) curve of Wilson in the left side.

A 23-year-old male patient presenting a Class III malocclusion with a lateral deviation of the mandible to the left side associated with a cross-bite of teeth 2.2, 2.3, and 2.4 came to our attention (Figure 1). Articular dysfunction in both Temporomandibular Joints (TMJs) was present, particularly focused on the left side. On the working side it was possible to observe a shorter and thicker condyle and mandibular ramus; conversely, in the opposite side, both areas appeared longer and thinner. Furthermore, it was possible to notice a steeper articular eminence on the left side, associated with a more posterior position of the condyle, responsible for symptoms such as pain and articular dysfunction (Figure 2). Spee and Wilson curves were more accentuate on the left side, where a reduced dental and articular vertical dimensions could be observed (Figure 3). As a consequence, Spee and Wilson curves underwent a remodelling to compensate the loss of posterior occlusal contacts. In the present case, both skeletal and dental asymmetries are presented. The cephalometric analysis highlighted a brachyfacial type with a negative convexity associated with a slight tendency to Class III, even in the presence of a normal (Xi-PM) value (Table 1). The negative convexity value was related to the mandibular shifting toward the front and the right, due to the left cross-bite. The horizontal position of the maxilla (Pf-Na-A) showed a normal value (Figure 4). Thus, since this was not a real skeletal Class III, the surgical approach was performed in order to correct only the skeletal asymmetry developed during the years.

### 2.2. Treatment

In the present case, the orthodontic presurgical phase was performed using the Invisalign device [6–8].

**Figure 5:**
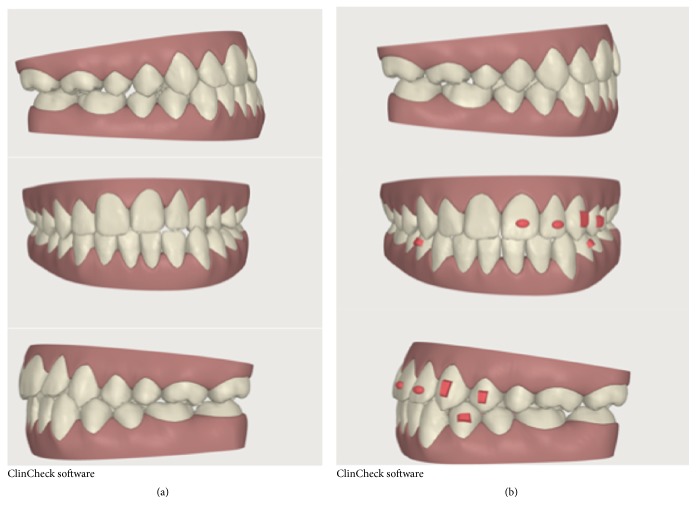
(a) ClinCheck pretreatment; (b) ClinCheck surgical simulation.

**Figure 6:**
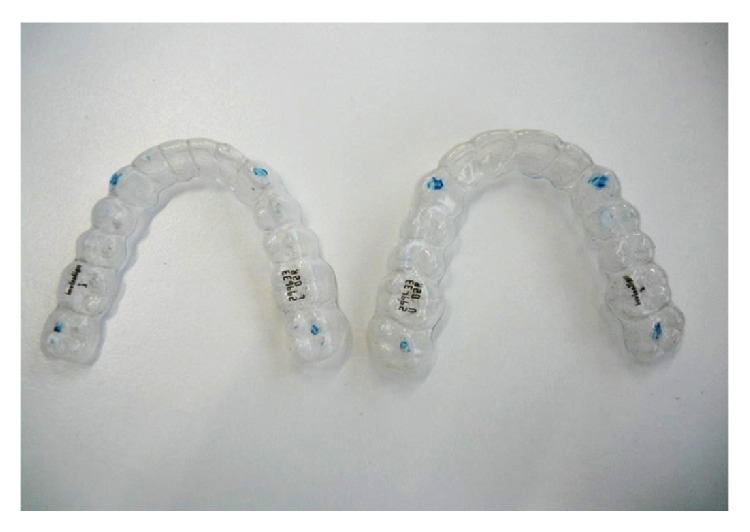
Clear orthodontic aligners.

**Figure 7:**
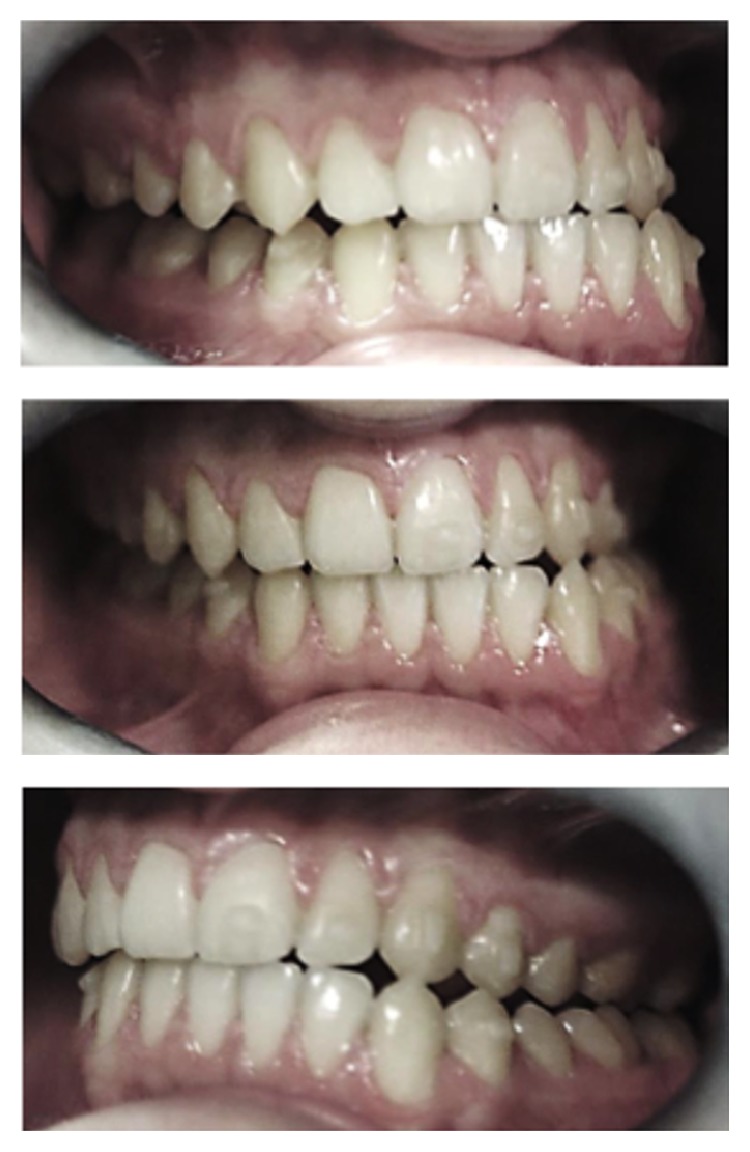
Clinical situation at the end of the orthodontic correction.

It was possible to previsualize the project and to plan each phase of the treatment, including the surgical correction, using the software ClinCheck® (Align Technology, Inc., San Josè, California, USA) [9] (Figure 5). The same software was also used to carefully evaluate the asymmetry of the dental arches, the occlusion, and the Spee and Wilson curves using different projections. The use of ClinCheck improved the quality of the diagnosis and allowed to specify the required dental movements in detail. Even in this planning phase, the clinician knowledge with regard to the software functions has an essential role. Actually, any kind of required modification can be applied within the software with the aid of a well-structured setup. Moreover, it is possible to require attachments and Interproximal Reduction (IPR) device systems to improve the precision of the dental movements. Dental movements are performed by a specific series of detailed aligners (Figure 6). In this case, a series of 19 aligners for the upper dental arch and 9 aligners for the lower dental arch were used. The aligners were applied by the patients 22 hours per day and changed every 15 days. At the end of the presurgical phase, after 10 months from the beginning, dental impressions were taken, the stone study casts were mounted on an articulator, and a simulation of the surgical movements, consisting of derotation and backward translations, was performed. Once the achievement of a correct occlusion was obtained (Figure 7), the day before the surgical operation, brackets were applied on teeth in patient's dental arches.

**Figure 8:**
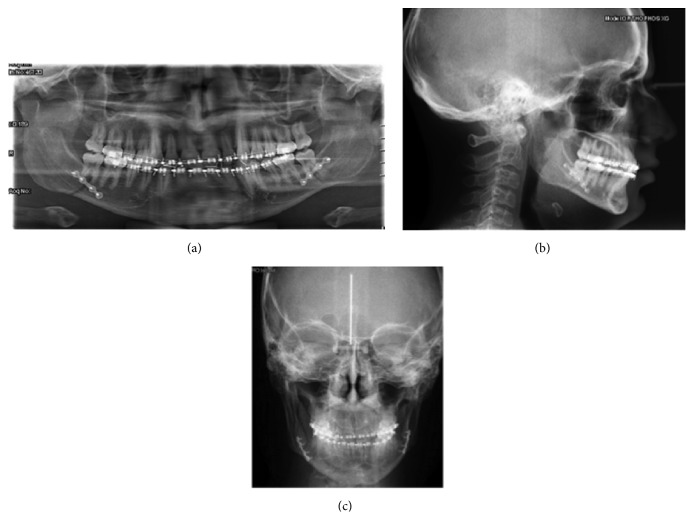
Postoperative radiological evaluation. (a) Orthopantomograph; (b) laterolateral teleradiography of the skull; and (c) teleradiography of the skull in a posteroanterior projection.

The surgical operation consisted in a bilateral sagittal split osteotomy with the application of titanium plates (Figure 8).

**Figure 9:**
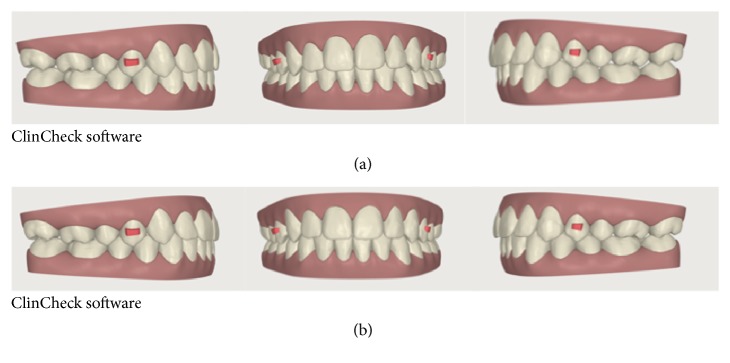
Finishing and detailing phase. (a) Beginning and (b) end of the procedure.

**Figure 10:**
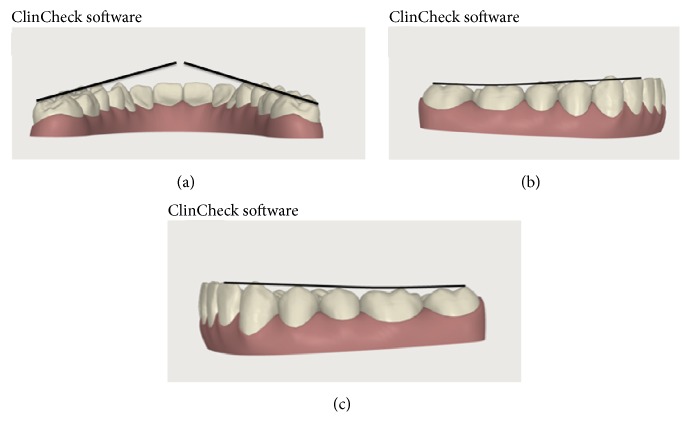
Curve of Wilson and Spee at the end of the finishing and detailing phase. (a) Curve of Wilson; (b) curve of Spee in the right side; and (c) curve of Wilson in the left side.

**Figure 11:**
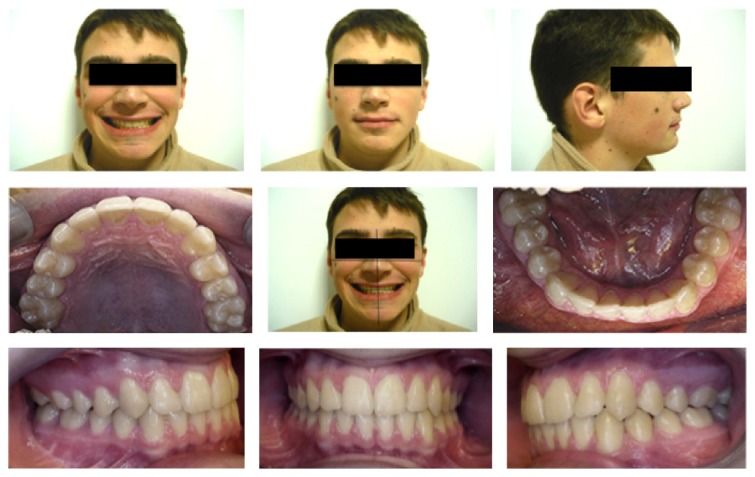
Clinical pictures of the patient at the end of the surgical and orthodontic treatment.

**Figure 12:**
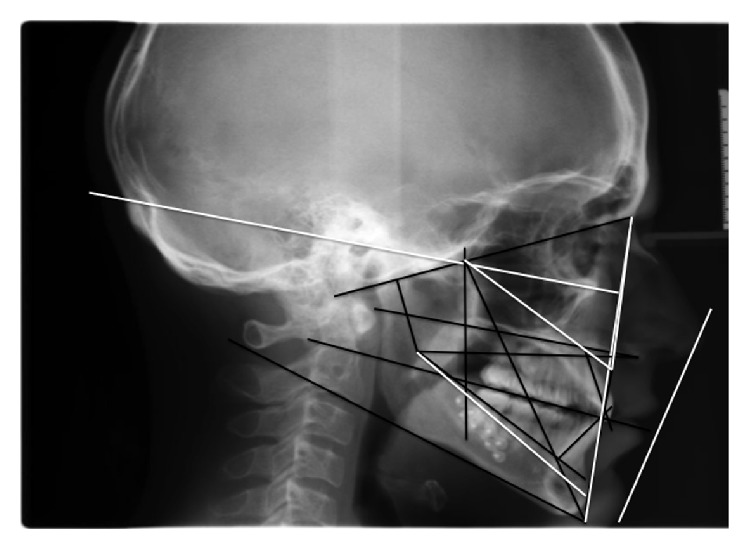
Laterolateral teleradiography of the skull and cephalometric analysis at the end of the treatment.

After one month, the brackets were removed and new dental impressions were taken, in order to start the postsurgical orthodontic phase with the following series of aligners. This consisted in a total of 5 aligners' series for both the upper and the lower dental arches (Figures 9 and 10). The whole treatment, including the pre- and postsurgical orthodontic phase, required 12 months (Figure 11). The posttreatment cephalometric analysis (Figure 12) showed an improvement of the maxillary vertical position (Na-CF-A), the maxillary orientation with respect to the horizontal plane (parallelism between FP-PNS-ANS planes), and the distance between lower lip-E (Table 1). The cephalometric values achieved after the treatment and maintained after 6 years showed a general enhance of the facial profile. The correction is evidenced especially by the variation of the facial axis and convexity values. Slight changes have been observed also for other values as evidenced in Table 1. However, the correction of the asymmetry is not fully appreciable by the cephalometric analysis due to its two-dimensional sagittal nature.

**Figure 13:**
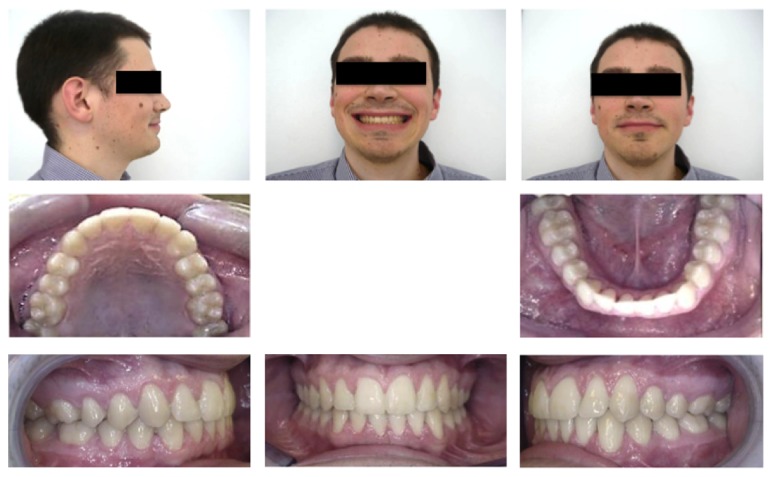
Clinical pictures of the patient at the 6-year follow-up recall visit.

**Figure 14 fig14:**
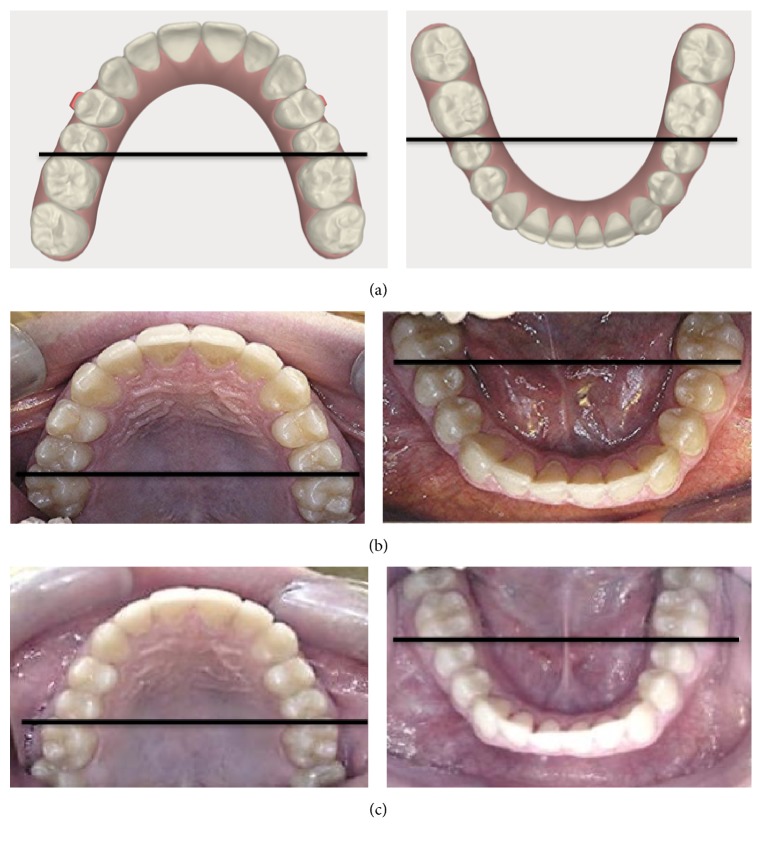
Posttreatment comparison of the interarch symmetry. (a) Clinical check evaluation at the end of the treatment; (b) clinical evaluation at the end of the treatment; and (c) 6-year follow-up clinical evaluation.

**Figure 15 fig15:**
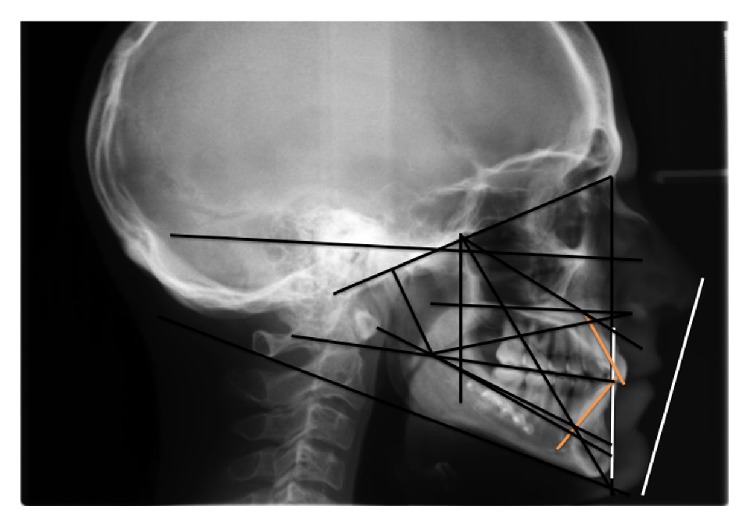
Laterolateral teleradiography of the skull and cephalometric analysis at the 6-year follow-up evaluation.

**Figure 16:**
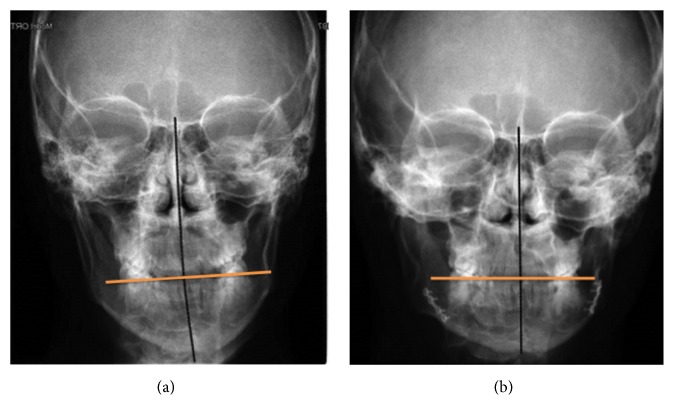
Comparison of the skull teleradiography in a posteroanterior projection before the treatment (a) and at the 6-year follow-up evaluation (b).

The follow-up at 6 years (Figures 13 [Fig fig14] [Fig fig15]–16) showed how dental and skeletal symmetry corrections, occlusion and functional balance, are stable over the time. Furthermore, in the following years, the patient reported a significant reduction of articular dysfunction, as well as the absence of pain.

## 3. Discussion

According to Planas, the patient developed a chewing system mostly or exclusively on the left side, defined as working side, whereas the right side was defined as the balancing side [10, 11]. In this type of situation, the morphologic mandibular development in length and in width, through the years, was oriented to a different growth of the two sides of the jaw, causing a skeletal asymmetry [12]. According to Deshayes, skeletal and subsequently dental asymmetries recognize a precise origin: the growth of the jaws depends on the growth trajectories of the skull bones, characterized by axial rotations and translational movements in relation to each others [13, 14]. These movements produce a flexion of the skull base that increases transversally and reduces the sagittal dimension. The skull base flection is the essential condition to gain a correct physiologic chewing function in order to let the children start to eat solid food, around 3 years, in association with an optimal mandibular functionality. Often, at this age, the correct development of an effective chewing function is not yet reached [15]. A skull base too flexed leads the mandible forward, tending to a prognathism condition (Class III). On the other hand, a too slow flection leads the mandible backward, with a consequent reduced chewing functionality. In this process, the conformation of the TMJs is also involved [16]. To reach an optimal and physiological chewing function, it is necessary to begin the therapy before the end of the skull base growth within the age of 6 [17, 18]. In the adult patient characterized by a mandibular prognathism and asymmetry, an orthodontic-surgical therapy will be necessary to reestablish the symmetry of the jaws, since it is not possible to exploit the growth pattern of the cranial bones anymore. During the diagnostic phase, a nonsurgical therapy might be considered, in order to reposition the mandible by means of a mouth guard, followed by an interarch dental reposition to reach a functional occlusion. This less invasive solution is not always able to solve the typical problematic of a severe morphologic-skeletal asymmetry, but it might be useful in less extreme cases, such as functional asymmetries.

Several authors have described different advantages and limitations when using Invisalign and similar systems. Its application has been successfully reported in the treatment of Class III, molar distalization and premolar derotation [19–21]. A systematic review investigated the effectiveness of this kind of devices, showing indications and limits [22]. The usefulness in controlling anterior intrusion but not anterior extrusion has been observed; it is effective in controlling posterior buccolingual inclination but not anterior buccolingual inclination; it is indicated in controlling upper molar bodily movements of about 1.5 mm, but it is not effective in controlling rotation of rounded teeth in particular. Furthermore, the use of Invisalign has been also related to a better periodontal health and, according to our paper, to a better patient's satisfaction [23].

The present paper shows the possibility to use an alternative device instead of the traditional fixed appliance before and after orthognathic surgery. Invisalign provided accurate and precise results guaranteeing a better aesthetic, the maintenance of oral hygiene, and a comfortable management of the removable appliance. Furthermore, patient satisfaction was recorded as very high due to the invisible orthodontic treatment, and, above all, his occlusion was functionally rehabilitated.

## Figures and Tables

**Table 1:** Cephalometric analysis. Data before and at the end of the treatment. After a follow-up period of 6 years no variations were observed.

	Normal values (±SD)	Growth variation	Before treatment	After treatment
Facial axis	90° (±3°)	0°	96°	94°
Facial angle	87° (±3°)	±1°/3 years	93°	94°
Frankfort mandibular plane angle	26° (±4°)	−1°/3 years	20°	21°
Lower facial height	47° (±4°)	0°	43°	44°
Mandibular arch angle	26° (±4°)	+0.5°/year	31°	36°
Convexity	+2 mm (±2 mm)	−1 mm/3 years	−1.5 mm	+2 mm
Na-CF-A	54° (±3°)	+1°/3 years	63°	56°
PF-Na-A	90° (±3°)	—	89°	95°
PF-bispinal plane	1° (±3°)	—	−5°	1°
Xi-Pm	66 mm^2^ (64–70 mm)	—	65 mm	67 mm
Li-APo distance	1 mm (±2 mm)	0 mm	+2 mm	1 mm
Ls-APo distance	4 mm (±2 mm)	—	+3 mm	+3 mm
Li-APo angle	22° (±4°)	0°	30°	34°
Interincisal angle	130° (±6°)	—	123°	121°
PTV-U6	Age + 3 mm (±2 mm)	1 mm/year	8 mm	13 mm
Overjet	2.5 mm (±2 mm)	—	+1 mm	+3 mm
Overbite	2.5 mm (±2 mm)	—	0 mm	+2 mm
Li-E distance	−2 mm (±2 mm)	—	−8 mm	−4 mm
